# Synergistic benefits of melatonin and therapeutic exercise as a unified strategy for managing stroke and doxorubicin-induced cardiotoxicity

**DOI:** 10.3389/fneur.2025.1567263

**Published:** 2025-11-05

**Authors:** Sookyoung Park, Zeeshan Ahmad Khan, Yunkyung Hong, Jinhee Shin, Tserentogtokh Lkhagvasuren, Seunghoon Lee, Yonggeun Hong

**Affiliations:** ^1^Department of Physical Therapy, College of Healthcare Medical Science & Engineering, Inje University, Gimhae, Republic of Korea; ^2^Research Center for Aged-Life Redesign (RCAR), Inje University, Gimhae, Republic of Korea; ^3^Biohealth Products Research Center (BPRC), Inje University, Gimhae, Republic of Korea; ^4^Department of Physical Therapy, Graduate School of Inje University, Gimhae, Republic of Korea; ^5^Department of Smart Foods & Drugs, Graduate School of Inje University, Gimhae, Republic of Korea; ^6^Department of Rehabilitation Science, Graduate School of Inje University, Gimhae, Republic of Korea

**Keywords:** stroke, melatonin, exercise, doxorubicin, chemotherapy

## Abstract

**Introduction:**

Epidemiological studies highlight a significant occurrence of ischemic strokes in cancer patients, particularly in the elderly, where stroke and systemic cancer are the leading causes of death. This comprehensive study investigates the combined effect of melatonin (MT) and exercise (Ex) against stroke, and a chemotherapeutic drug doxorubicin (Dox)-induced cardiotoxicity (DIC).

**Method:**

This study employs two models—middle cerebral artery occlusion (MCAo) and DIC—to evaluate the synergistic effects of MT + Ex. For both models, rats were assigned to the same treatment groups (*n* = 5 per group): control, vehicle, MT (10 mg/kg, twice daily), Ex (10 m/min for 30 min/day), and combined MT + Ex after either MCAo or DIC. The treatments were administered for 4 weeks. Significance was determined using one-way ANOVA.

**Results:**

In the MCAo model, the MT + Ex group showed a significant reduction in brain infarct volume and neurological deficits compared to control animals (*p* < 0.01). Western blot analysis revealed downregulation of molecular markers associated with neuronal damage and enhanced neuronal growth in the treatment group (*p* < 0.01). Additionally, the MT + Ex group exhibited a higher density of dendritic spines and a progressive increase in neurite crossing at days 14 and 28, compared to both MT and Ex groups (*p* < 0.01). In the DIC model, both MT and Ex treatments provided cardioprotection, while the combined MT + Ex group demonstrated a significantly greater protective effect against heart weight loss and histopathological damage compared to controls (*p* < 0.01). Also, pretreatment of MT significantly improves the cell viability compared to both Veh and resveratrol but its effect on beating frequency of cardiomyocytes was similar to resveratrol.

**Conclusion:**

MT + Ex reduced brain infarct volume and neurological deficits, enhanced neuronal growth, and provided superior cardioprotection, preventing heart weight loss and histopathological damage. This study is the first to show synergistic protective action of MT and Ex against stroke and DIC, contributing key insights into an interplay between neurological and cardiovascular health and addressing multifaceted challenges posed by stroke, and anticancer interventions.

## 1 Introduction

Stroke and cancer pose significant health challenges, especially among older individuals. A recent study explored this connection, revealing that out of 7,529,481 cancer patients, 80,513 tragically passed away from fatal strokes (1). This study highlighted a higher risk of deadly strokes in cancer patients, showing a mortality rate of 21.64 per 100,000 person-years and a standardized mortality ratio (SMR) of 2.17 (95% CI, 2.15, 2.19) (1). Patients with cancer face an increased likelihood of strokes due to the tumor’s direct effects, blood clot issues, and the treatments they undergo. Interestingly, one in seven to eight patients with ischemic stroke are found to have known or hidden cancer (2). Cancer chemotherapy, especially in individuals with a history of stroke, can lead to severe and life-threatening side effects. Current therapeutic options for both conditions are limited, emphasizing the need for strategic interventions to aid stroke recovery and complement cancer chemotherapy. This approach has significant potential benefits for patients dealing with both conditions.

Doxorubicin (Dox), commonly known as the “red devil” and a potent antineoplastic agent, plays a crucial role in multidrug chemotherapy against various cancers (3). Despite its effectiveness in reducing cancer-related mortality, Dox is associated with severe cardiac effects, including late-onset cardiomyopathy, left ventricular dysfunction, and heart failure (4). Chemotherapy-related cardiotoxicity can manifest acutely, subacutely, or chronically, posing challenges in cancer treatment. Oxidative stress, inflammation, mitochondrial impairment, apoptosis, and dysregulation of autophagy contribute to Dox-induced cytotoxicity (DIC) (4). While alternative chemotherapeutic agents like trastuzumab may also have adverse effects, there is currently no equivalent substitute for Dox, making it a first-line therapy for several malignancies (5). Given the widespread occurrence of DIC, extensive research has explored various strategies to mitigate these effects. *In vivo* pharmacological interventions, including renin-angiotensin system antagonists, anti-diabetic drugs, and late Na^+^ current blockers, show potential, but limitations exist (6). Modifying Dox delivery through formulations like Doxil^®^ and nanocomposites has demonstrated reduced cardiotoxicity, yet challenges persist, including increased cardiac risk with dosage (5). Novel strategies are urgently needed to address Dox’s cardiotoxicity, emphasizing the search for biocompatible, cost-effective, and easily synthesized biomolecules for more effective and safer cancer treatments.

In mammals, melatonin (MT) is naturally synthesized from the amino acid tryptophan, primarily under the influence of the hypothalamic suprachiasmatic nuclei, which receive daily light and dark patterns from the retinohypothalamic tract (7). Pineal melatonin, synthesized in the pineal gland, primarily regulates circadian rhythms and is typically present in nanomolar concentrations (7, 8). In contrast, extrapineal is from multiple tissues throughout the body, probably being synthesized in the mitochondria of these cells, which constitute the bulk of the melatonin produced in mammals and is concerned with metabolic regulation (9). Extrapineal melatonin exhibits crucial antioxidant, anti-inflammatory, and mitochondrial protective effects Pineal melatonin, synthesized in the pineal gland, primarily regulates circadian rhythms and is typically present in nanomolar concentrations (7, 8). In contrast, extrapineal is from multiple tissues throughout the body, probably being synthesized in the mitochondria of these cells, which constitute the bulk of the melatonin produced in mammals and is concerned with metabolic regulation (9). Extrapineal melatonin exhibits crucial antioxidant, anti-inflammatory, and mitochondrial protective effects Pineal melatonin, synthesized in the pineal gland, primarily regulates circadian rhythms and is typically present in nanomolar concentrations (7, 8). In contrast, extrapineal melatonin is produced in many peripheral tissues, most likely within cellular mitochondria, and constitutes the majority of melatonin in mammals, where it chiefly regulates metabolism (9). This extrapineal melatonin is shown to demonstrate potent antioxidant, anti-inflammatory, and mitochondrial-protective effects (9). However, pan-pinealectomy in mammals eliminates plasma melatonin and its daily rhythm, leaving the relative contribution of extrapineal sources unclear (9, 10). Acting as a major secretory product of the pineal gland, MT has been recognized for its neuroprotective role in neurodegenerative diseases such as Parkinson’s disease, Alzheimer’s disease, and stroke (11). Its antioxidant properties serve as a potent free radical scavenger during ischemic/reperfusion injury, contributing significantly to neuroprotection. Studies show that MT treatment before reperfusion reduces brain damage in focal cerebral ischemic rats and promotes neurogenesis, addressing short-term memory impairments (12). Similarly, exercise (Ex) training induces heart antioxidant systems, mitochondrial function, and protection against myocardial insults like ischemia and reperfusion (13). Considering the opposing behavior of MT and Ex against Dox-induced cardiomyopathy, a synergistic approach of MT and Ex emerges as a potential complementary treatment to Dox chemotherapy. This highlights the need for a study to assess the protective activity of MT and Ex against Dox-induced cardiomyopathy, presenting a promising avenue for enhanced therapeutic strategies.

This study delves into a novel approach for stroke and DIC. We hypothesize that the combined treatment of MT and Ex (MT + Ex) will have a synergistic effect, leading to improved functional recovery, enhanced neurite outgrowth, and better neural circuit reorganization in a focal cerebral ischemic rat model, as well as superior cardioprotection against DIC compared to individual treatments of MT or Ex. By elucidating the synergistic effects of MT + Ex, we aspire to contribute to a deeper understanding of post-stroke neurorehabilitation strategies, offering insights that could lead to more effective interventions and significantly enhance the quality of life for stroke survivors. This multifaceted investigation aims to uncover key insights that bridge the gap between stroke recovery and cardioprotection.

## 2 Materials and methods

### 2.1 Ethics statement

All animal procedures were approved by the Ethics Committee for Animal Care and Use of Inje University (Approval No. 2021-009, 2022-006), which is certified by the Korean Association of Accreditation of Laboratory Animal Care. Food and water were available *ad libitum*. Effects were taken to minimize the pain of animals. Experiments were performed following ARRIVE guidelines.

### 2.2 Induction of focal cerebral ischemia/reperfusion

**Figure 1:**
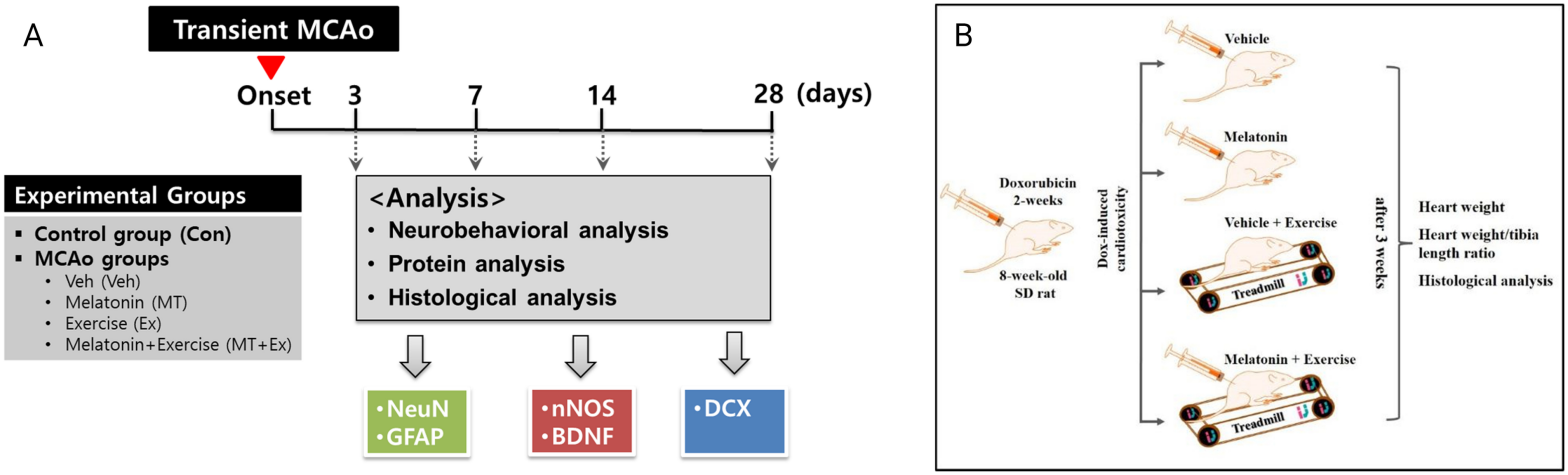
**(A)** Experimental design and animal groups in present study. This *in vivo* study followed the scheme shown here, which is summarized in “materials and methods.” Transient MCAo: Transient middle cerebral artery occlusion. Veh and control were considered same in TTC staining to reduce the number of animals used in this study. **(B)** Schematic diagram of experimental design. Dox-induced cardiotoxic animals were randomly divided into four groups, vehicle control, melatonin, exercise, and exercise + melatonin. Heart weight, heart weight/tibia length ratio, and histological analysis were performed after 5 weeks of treatment.

Male Sprague–Dawley rats (8-week-old, 240–270 g) were purchased from Daehan Bio Link (Hyochang Science, Daegu, Korea) and were individually housed in plastic cages under controlled temperature (22 ± 1 °C), relative humidity (55 ± 10%) and established photoperiod (light/dark conditions 12/12 h; lights on 07:00 a.m.). To induce focal cerebral ischemia, intraluminal suture middle cerebral artery occlusion (MCAo)/reperfusion micro-surgery was conducted on rats as previously described (14). Experimental rats were randomly divided into five groups (*n* = 5 for each group): control (Con) and middle cerebral artery occlusion (MCAo) groups treated vehicle (Veh, 1% ethanol in saline), MT (10 mg/kg body weight in 1% ethanol, MT), Ex (Ex; 10 m/min, 30 min/day, 1% ethanol in saline twice) and MT with Ex (MT + Ex) for 4 weeks. The body mass measurements were taken on a daily basis to ensure accurate adjustments in drug administration. The experimental procedures of this study are illustrated in Figure 1A.

### 2.3 Interventions of melatonin and exercise

The interventional protocols for MT and Ex based on previous studies (15–19). Melatonin (Sigma-Aldrich. St. Louis, MO, United States) was injected subcutaneously at a dose of 10 mg/kg twice a day (Dawn at 07:00, Dusk at 19:00) for 4 weeks. Concurrently, moderate treadmill Ex was applied at 20 m/min, for 30 min/day, for five consecutive days. Before initiating the formal Ex regimen, all rats assigned to the Ex and MT + Ex groups underwent a 5-day adaptation period. During this phase, animals were placed on the treadmill for gradually increasing durations and speeds, starting at 5 m/min for 10 min on day one and progressing to 10–15 m/min for 20–30 min by day five. This approach allowed the animals to become familiar with the treadmill environment and running behavior, minimizing stress and ensuring consistent performance during the experimental period. All the exercise regimens were performed in a well-lit room during the wakefulness period of animal at the same time every day.

### 2.4 Evaluation of neurological deficit

Neurological deficiency and motor dysfunction induced by focal cerebral ischemia were evaluated by modified Neurological Severity Score (mNSS). The mNSS is a composite of motor, sensory, reflex, and balance tests, and it shows neurological deficiencies graded on a scale of 0 to 18 (normal score = 0; maximal deficit score = 18). The behavioral test was performed on days 1, 3, 7, 14, and 28 after MCAo by an investigator who was blinded to the experimental groups.

### 2.5 Measurement of cerebral infarct volume

2% 2,3,5-Triphenyltetrazolium chloride (TTC) staining was commonly used to determine the cerebral infarct volume. Animals were subjected to MCAo and sacrificed at different time points (14). The brain was removed immediately and dissected into 2-mm-thick coronal sections from +4.00 to −4.00 relative to the bregma for analysis of the infarct volume using a rat brain slicer (Rodent Brain Matrix, Adult rat, Coronal Sections, ASI Instruments, Warren, MI, United States). Brain slices were immersed for 30 min in 2% TTC (Sigma–Aldrich, St. Louis, MO, United States) in PBS at 37 °C with light protection. Subsequently, stained sections were post-fixed in 10% buffered formalin overnight and scanned by a photo scanner. The infarct volume was measured with the set scale function of the Image J program (ver. 1.6, NIH, Bethesda, MD, United States) as described previously (20–22). For exact quantification of infarct volume, excluding brain tissue edema, the infarct volume was based on the contralateral hemisphere area.

### 2.6 Protein extraction and Western blotting

The Ipsilateral hemisphere was isolated from the whole brain tissue of the experimental animals and used for western blotting (14). Specimens were homogenized in ice-cold lysis buffer supplemented with a protease inhibitor cocktail (Roche, Penzberg, Germany) to extract protein. The concentration of protein was estimated by the Bradford assay (Bio-Rad Laboratories, Richmond, CA, United States) using a spectrophotometer. Protein samples containing 20 μg were separated on SDS-PAGE and transferred to a PVDF membrane (Merck Millipore, Billerica, MA, United States). These blots were probed with following primary antibodies and incubated at 4 °C overnight: rabbit polyclonal anti-NeuN (EMD, Millipore), goat polyclonal anti-DCX, goat polyclonal anti-BDNF, and rabbit polyclonal anti-GAPDH (Santa Cruz Biotechnology, United States), rabbit polyclonal anti-GFAP, rabbit polyclonal anti-nNOS (Cell Signaling Technology, Danvers, MA, United States). Membranes were incubated with the secondary antibody. Specific bands were visualized with enhanced chemiluminescent (ECL) reagents (SuperSignal, Thermo Scientific, Rockford, IL, United States) and quantified using the Image J software.

### 2.7 Golgi-cox staining

The rats were euthanized by decapitation. Subsequently, the brains of experimental animals were stained by the Golgi-cox method using the FD NeuroTechnologies Rapid GolgiStain^™^ kit (FD NeuroTechnologies, Ellicott City, MD, United States) according to the manufacturer’s protocol (14). The brain tissues were immersed in silver impregnation solution for 2 weeks in the dark and followed by immersion in solution C for 1 week. The stained tissue was serially sectioned to 200 μm-thick using a cryostat microtome and mounted onto gelatin-coated slides. These brain sections were dried at room temperature before staining with Solutions D and E from the kit. Sections were dehydrated using an alcohol series and clarified using xylene before cover-slipping with Permount (Fisher Scientific, Pittsburgh, PA, United States). Slices were analyzed with an OLYMPUS DP70 microscope digital camera (Olympus, Tokyo, Japan) connected to a computer. The number of dendritic spines was normalized to the control for a 10-μm segment length. The spine density represents the number of spines/μm in all groups.

### 2.8 Neonatal cardiomyocyte primary culture

Primary cultured cardiomyocytes were obtained from neonatal Sprague–Dawley rats using a Neonatal Cardiomyocyte Isolation System (Worthington Biochemical Corporation, Lakewood, NJ, United States). Neonatal rats (up to 3 days postnatal, *n* = 15) were rinsed in ethanol solution for sterilization and surgically removed from the beating heart. Neonatal hearts were extracted from the body with micro-scissors and transferred immediately in the sterile tube containing phosphate buffer saline (PBS; Lonza, Basel, Switzerland). The tube was swirled to rinse hearts and was transferred in sterile calcium- and magnesium-free Hank’s balanced salt solution (CMF HBSS, Worthington, United States).

The tissue was minced with small scissors or a razor blade into a 1 mm^3^ piece at 0 °C. Minced tissue was supplemented with trypsin and placed in the refrigerator overnight (16–20 h) at 2–8 °C. The tissue was supplemented with the trypsin inhibitor warmed to 37 °C in the water bath, and then transferred to calcium-containing medium buffer. The tube containing suspended cardiomyocytes was placed on a shaker at 37 °C for 30–45 min (2–4 rpm). After this procedure, the buffer containing the tissue was triturated about 10 times with a standard 10 mL plastic serological pipette. The cell strainer was rinsed with 1 mL of the L-15 culture medium (Leibovitz L-15 medium, Worthington, United States). The tissue was filtered the supernatant t through the cell strainer into a fresh 50 mL sterile tube. And the tissue was added 5 mL additional L-15 to filter the tissue residue and processed trituration step repeatedly. The cells were plated in the cell culture dish for 1 h to allow the remaining fibroblasts to attach. Unattached cardiomyocytes were then plated in 6-well plates covered with gelatin in a culture medium M199 (Medium 199, Lonza, Basel, Switzerland) supplemented with 10% FBS, penicillin/streptomycin (60 μg/mL).

### 2.9 Treatment of primary cardiomyocytes with melatonin and resveratrol, and cell viability assay

To assess cell viability, isolated cultured primary cardiomyocytes were plated at a density of 5 × 10^4^ cells/cm^2^ in 96-well plates. Pre-and post-treatment experiments were performed with Veh, MT, and Resv. In the pre-treatment group, the cultured cells were treated with culture medium alone, or 1% ethanol Veh, or 1 μM Resv, or 1 μM MT for 12 h. Subsequently, 1 μM Dox was supplemented in all the groups. Whereas, in the post-treatment group, the cultured cells were treated with 1 μM Dox for 24 h followed by 12 h treatment with Veh, Resv or MT. After the 36-h treatment, the cells were washed twice with PBS and 20 μL 3-(4,5-dimethylthiazol-2-yl)-2,5-diphenyltetrazolium bromide (MTT; 5 mg/mL) was added to each well. The plate was incubated for an additional 4 h at 37 °C, and crystal formazan was dissolved by replacing the media with 150 μL dimethyl sulfoxide (0.1 mL/well). Absorbance at 490 nm was measured using a Fluorescence Multi-Detection Reader (BioTek, Winooski, VT, United States). Microphotographs and beating frequency analyses of the treated and control cardiomyocytes were performed before the addition of MTT. Viability and beating frequency were presented as percent differences and differences from the control samples. All the experiments were performed at least three times, the results are presented as the change from the mean ± standard error of the mean (S.E.M.).

### 2.10 Rodent model of doxorubicin-induced cardiotoxicity

Cardiotoxicity was induced in all rats by intraperitoneal injection of Dox (2.5 mg/kg body weight) three times per week. After Dox administration which lasted 2 weeks, the rats were randomly divided into four groups, each containing five rats: (1) Veh (1% ethanol in saline twice per day for 3 weeks), (2) MT (10 mg/kg body weight in 1% ethanol, twice per day), (3) Ex, exercise on a treadmill (10 m/min, 30 min/day, 1% ethanol in saline twice), and (4) MT + Ex (10 mg/kg body weight in 1% ethanol, twice per day 10 m/min, 30 min/day) for 4 weeks. For Ex group, the rats were placed on the treadmill, according to their running capabilities (7–10.5 m/min). The body mass measurements were taken on a daily basis to ensure accurate adjustments in drug administration. In case of animals were unable to plantar stepping, the hip was manually lifted to partially unload the hindlimbs, also, the trainer provides bodyweight support if necessary. If the rats were not stepping off the hindlimbs in response to the treadmill movements and the stepping of the forelimbs, manual stimulation was given to the perineum. All experiments lasted 5 weeks (2 weeks of inflammation followed by 3 weeks of treatment) (Figure 1B).

### 2.11 Histological analysis

The anesthetized rats were perfused transcardially with 0.1 M PBS (pH 7.4) and fixed with 4% neutral buffered paraformaldehyde (pH 7.4). The samples were embedded in Tissue-Tek^®^ OCT compound (Sakura Finetek, Torrance, CA, United States) after fixation in 4% neutral buffered paraformaldehyde (pH 7.4). Cryosections (10 μm thick) were prepared using an HM525 cryostat microtome (MICROM International GmbH, Walldorf, Germany). Tissue sections were subjected to hematoxylin and eosin (HE) staining. Each specimen was analyzed using an Olympus DP70 digital microscope camera (Olympus, Tokyo, Japan) connected to a computer. Data were collected from repeated experiments and are presented as means ± S.E.M.

### 2.12 Statistical analysis

For experiments on MCAo-stroke, data were collected from repeated experiments and were presented as mean ± SD. Significance was determined using one-way ANOVA with a post-hoc Duncan test. For DIC experiments, statistically significant differences between groups were assessed by Kruskal–Wallis analysis of variance and the Mann–Whitney *U*-test or ANOVA followed Tukey’s *post hoc* comparison, using the mean to account for unequal groups. The normality of data was assessed by Shapiro–Wilk test. The MCAo-stroke experiment used the Duncan test following ANOVA because data were normally distributed, and the test assumptions were met. Meanwhile, the DIC experiment utilized non-parametric methods like the Kruskal–Wallis and Mann–Whitney *U*-test to address non-normal distributions and unequal group sizes, ensuring robust and valid statistical comparisons under these conditions. Differences were deemed to be statistically significant when the *p*-value was <0.05. All analyses were performed using the statistical software SPSS software (ver. 20.0; IBM, New York, NY, United States).

## 3 Results

**Figure 2:**
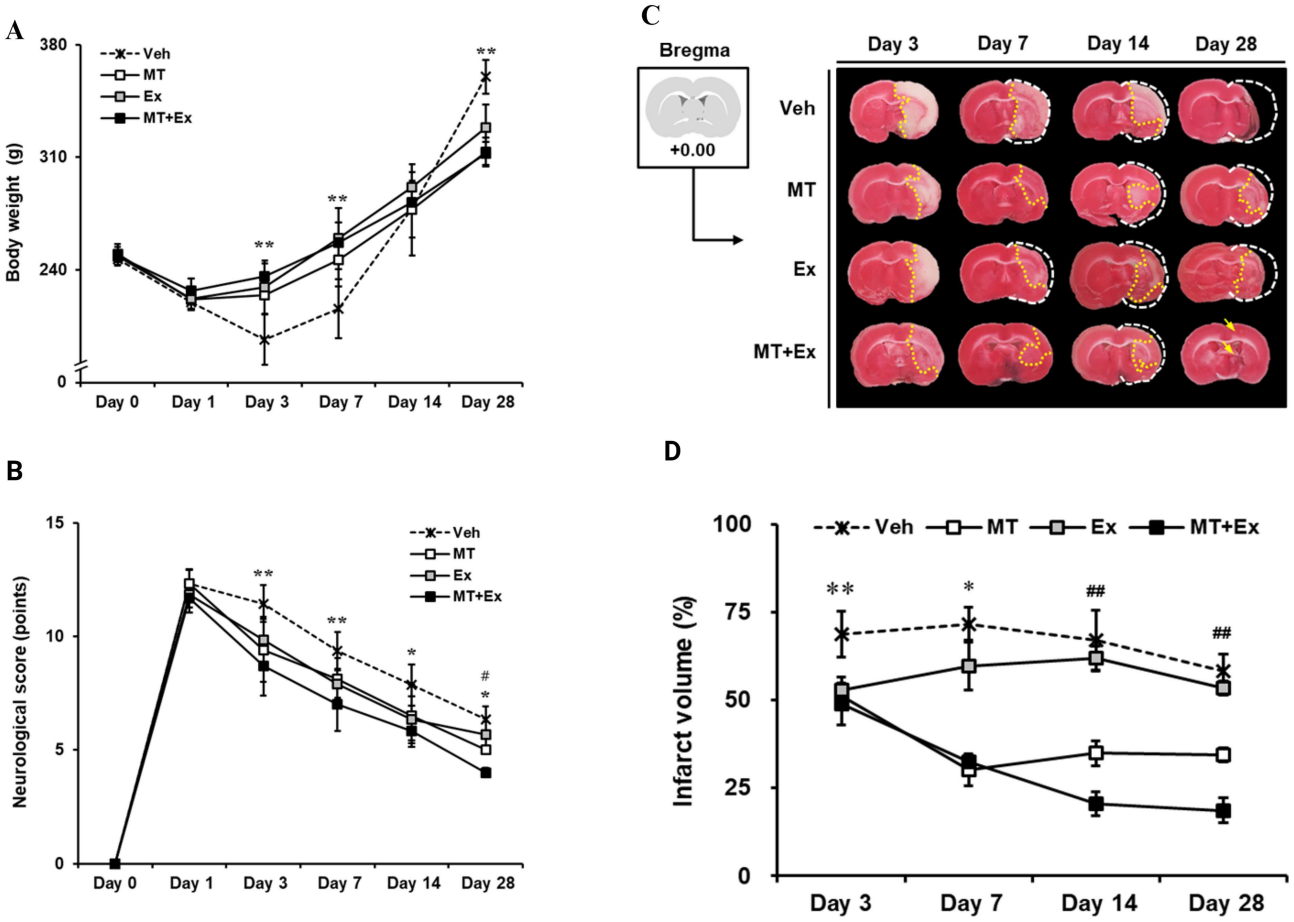
Sequential tracing of body weight and neurological deficits after cerebral ischemic injury. **(A)** Alterations of body weight were measured in experimental animals for 4 weeks after MCAo. ^**^p < 0.01 compared with Veh. Data shown are means ± SD. **(B)** Effect of melatonin and exercise on recovery of neurological behaviors. The degree of neurological deficiency was assessed by the mNSS criteria. **(C)** 2% TTC-defined ischemic infarct volume after MCAo. **(A)** TTC-stained images of experimental animal. The brain of the control model was intact; the white color on the right shows the infarction in the MCAo brains. **(D)** Quantification of infarct volume using Image J software. ^**^p < 0.01 and ^*^p < 0.05 compared with Veh; ^##^*p* < 0.01 compared with MT + Ex; data are presented as mean ± SD.

Body weight was traced periodically for analysis of physiological changes over 28 days after MCAo. All MCAo groups including Veh, MT, Ex, and MT + Ex, lost body weight immediately (20.2, 10.1, 7.7 and 5.1%, respectively) during the first 3 days after MCAo. However, MT, Ex and MT + Ex-treated animals showed a significant increase in body weight compared with Veh-treated rats until 7 days (*p* < 0.01). Subsequently, body weight gradually increased similarly in all groups for up to 14 days. At day 28 post-MCAo, Veh rats reached 146.0% of their initial weight; MT, Ex, and MT + Ex rats reached 125.4, 132.2, and 125.6%, respectively, (Figure 2A and Table 1).

**Table 1:** Effect of melatonin and exercise on alteration of body weight after MCAo injury.

Days	Veh	MT	Ex	MT + Ex
Day 0	246.6 ± 3.90	249.7 ± 6.53	248.4 ± 3.48	248.6 ± 5.45
Day 1	219.9 ± 5.03	221.7 ± 6.20	222.2 ± 6.39	226.9 ± 7.61^*^
Day 3	196.7 ± 15.51	224.5 ± 12.12^**^	229.2 ± 16.45^**^	235.9 ± 8.13^**^
Day 7	215.8 ± 18.48	246.1 ± 16.47^**^	259.4 ± 18.94^**^	257.1 ± 12.58^**^
Day 14	277.8 ± 17.51	277.4 ± 28.36	291.2 ± 9.47	282.0 ± 7.32
Day 28	360.0 ± 10.54	313.2 ± 8.83^**^	328.4 ± 14.43^**^	312.4 ± 7.16^**^
Day 0–3	20.2%	10.1%	7.7%	5.1%
Day 0–28	146.0%	125.4%	132.2%	125.6%

Data are shown as means ± SD (upper column), and percentage of decrease and increase from initial body weight (lower column) ^*^p < 0.05 and ^**^p < 0.01 compared with Veh.

mNSS test was conducted from 24 h until day 28 after MCAo surgery, and moderate injury (8–13 scores) was identified in all MCAo groups on the first day (Veh, 12.3 ± 0.6; MT, 12.3 ± 0.63; Ex, 11.8 ± 0.58; MT + Ex, 11.7 ± 0.63, respectively). We found MCAo rats showed typical neurologic deficiency and motor dysfunction at 24 h post-MCAo such as spontaneous circling to the paretic side, flexion of the paretic forelimb during tail suspension, and weak grip strength in the paretic forelimb. Thereafter, spontaneous recovery of function was observed over the next 4 weeks in all experimental groups, particularly, MT, Ex, and MT + Ex groups showed less neurological deficits compared with Veh groups at all time points after MCAo (*p* < 0.01 at day 3 and 7, *p* < 0.05 at day 14 and 28, respectively). In addition, the MT + Ex group exhibited a significant decrease in neurological deficit compared with any other treatment groups on day 28 after MCAo (*p* < 0.05). These results indicate that combined treatment of MT and Ex attenuated the severity of neurological damage significantly compared to a single treatment (Figure 2B).

To evaluate ischemic brain injury, the infarct volume was visualized by TTC staining (Figure 2C) and expressed as percentages relative to the contralateral hemisphere excluding areas of edema (Figure 2D). Lesion volumes were determined on the brain slice from +0.00 relative to the bregma at 3, 7, 14 and 28 days after MCAo surgery, respectively. On day 3, the ischemic lesions were observed mostly in the striatum, particularly Veh group showed expansion of the ischemic core from the striatum to the cerebral cortex. However, MCAo rats treated with MT and Ex presented lesions limited to the ischemic core, in addition to infarct volumes of the MT + Ex group were significantly less than other groups from day 3 to 28 after MCAo (*p* < 0.01). These results support that both MT and Ex have a neuroprotective effect on ischemic cerebral injury, furthermore MT combined with Ex attenuated neurological deficit efficiently compared to single treatment.

**Figure 3:**
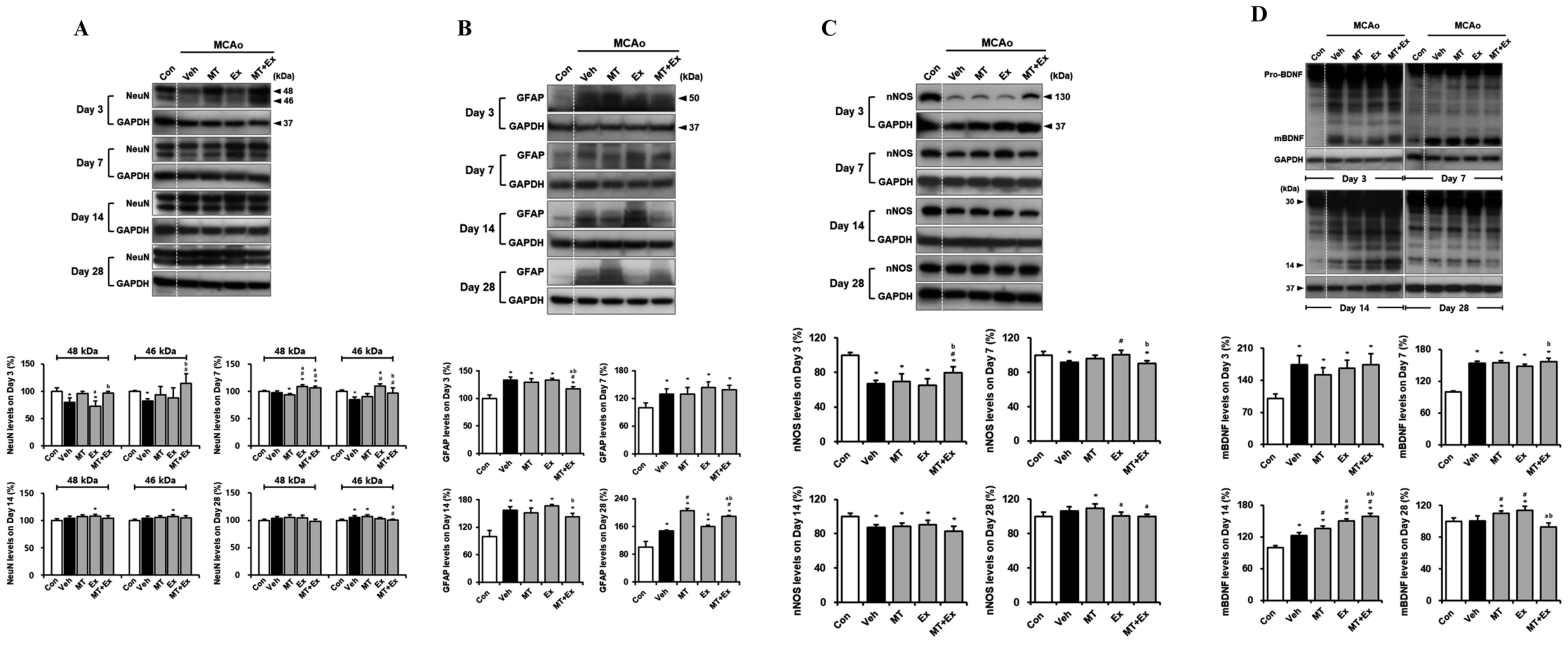
Downregulation of neuronal injury by melatonin plus exercise showing neuroprotective effects. **(A)** The expressions of 46- and 48-kDa of NeuN isoforms in the infarct region **(B)** GFAP **(C)** nNOS and **(D)** mBDNF expression was analyzed in the infarct region. ^*^p < 0.05 compared with Con; ^#^*p* < 0.05 compared with Veh; ^a^*p* < 0.05 compared with MT; ^b^*p* < 0.05 compared with Ex; data are presented as mean ± SD. The full images of western blots are given in Supplementary material 1.

To confirm whether MT and Ex switched the restoration machinery on for neurological function in MCAo rats, we performed the western blotting analysis using the target molecules for neuron and astroglia in damaged region of brain. We detected the two isoforms of NeuN, 46- and 48-kDa (Figure 3A). This both the 46- and the 48-kDa NeuN isoforms can be localized to the cell nucleus as well as in the neuronal cytoplasm, however, with the 48-kDa isoform being the predominant isoform in the cytoplasm (23). We quantified the protein levels from both 46- and 48-kDa NeuN isoforms separately (Figure 3A). The NeuN expressions decreased significantly in the Veh-treated rats in the first 3 days compared with control rats (*p* < 0.05), however, it remained intact in the MT + Ex group, particularly higher than in both MT and Ex groups (*p* < 0.05). Expressions of both NeuN isoforms were clearly ameliorated in the MT, EX, and MT + Ex groups compared with Veh group from 7 days after MCAo (*p* < 0.05). Continuously, NeuN expressions were recovered without significant differences within all experimental groups.

Specific marker of astrocyte, GFAP expression was determined in the lesion area after MCAo (Figure 3B). GFAP protein levels were persistently enhanced in all MCAo animals compared with control rats during the next 4 weeks (*p* < 0.05). However, interventions via MT and Ex significantly downregulated the GFAP levels, especially it was significantly decreased in MT + Ex groups compared with MT and Ex groups from day 3 after MCAo (*p* < 0.05). Furthermore, GFAP expression was the lowest in the Ex-group compared with MT and MT + Ex groups at day 28 post-injury.

Subsequently, we examined the effects of MT and Ex on neuronal regeneration and functional recovery in cerebral ischemic injury. To investigate whether both MT and Ex affect nitric oxide formation in brain following ischemic damage, we studied the influence of MT and Ex on nNOS expression in MCAo rats (Figure 3C). MCAo distinctly reduced nNOS expression, particularly at day 3 post-injury (*p* < 0.05), while dual treatment of MT and Ex reversed this change (*p* < 0.05). nNOS expression then recovered continuously by MT and Ex from day 7, and it reached a peak on day 28 after MCAo. It suggests that MT plus Ex may enhance the formation of nitric oxide through up-regulation of nNOS in the infarct region of MCAo rats.

Next, we evaluated the BDNF protein level and whether ischemic injury would induce the alteration in BDNF expression, including immature precursor pro-BDNF and mature BDNF (mBDNF) (Figure 3D). We found no differences of 30-kDa of pro-BDNF expression within the experimental groups. However, 14-kDa of mBDNF expression was significantly amplified in response to ischemic brain injury compared with control rats until day 14 after MCAo (*p* < 0.05). All experimental groups then showed the reduction of mBDNF levels at day 28 post-injury, particularly it was significantly downregulated in MT + Ex group compared with both MT and Ex groups (*p* < 0.05).

**Figure 4:**
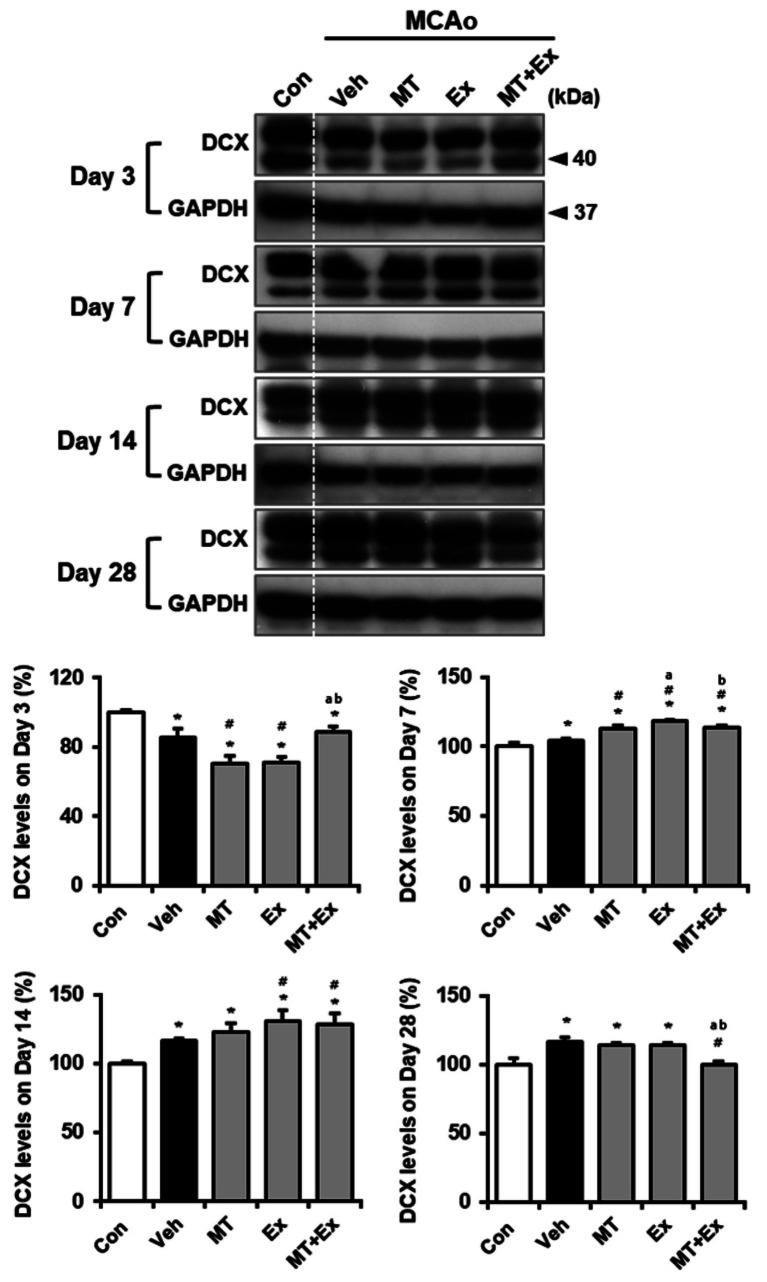
Melatonin and exercise promote neuronal migration and differentiation after ischemic insult. DCX expression was determined in infarct region of MCAo rats. ^*^p < 0.05 compared with Con; ^#^*p* < 0.05 compared with Veh; ^a^*p* < 0.05 compared with MT; ^b^*p* < 0.05 compared with Ex; data are presented as mean ± SD. The full images of western blots are given in Supplementary material 1.

To determine whether neuronal migration was promoted by MT plus Ex after ischemic brain injury, we analyzed the expression of Ex-reactive specific neuronal cell marker, DCX in MCAo rats (Figure 4). DCX protein level was significantly diminished by MCAo compared with control rats (*p* < 0.05), however it was attenuated in MT + Ex group at day 3 post-injury. DCX expression was then constantly increased by treatment MT and Ex until day 14 (*p* < 0.05), then its level was regulated by MT plus Ex at day 28 (*p* < 0.05).

**Figure 5:**
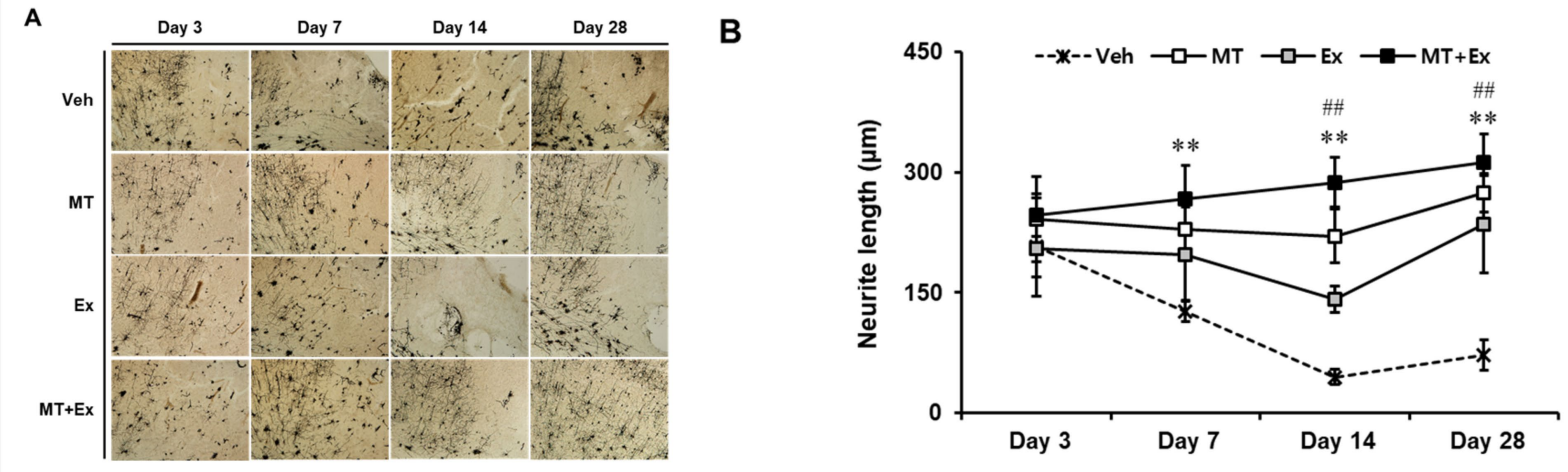
Golgi-cox-stained sections of the MCAo brain. **(A)** Region of analyzed neurons in the ipsilateral cortex. **(B)** Quantification of neurite outgrowth. Magnification = 400×. Scale bars = 500 μm. ^**^p < 0.01 compared with Veh, ^##^*p* < 0.01 compared with MT + Ex, data are presented as mean ± SD.

The dendritic spine is the major postsynaptic site of glutamatergic synapses, and plays a critical role in synaptic transmission, synaptogenesis and synaptic regulation. Golgi-cox staining is a key method to label a subset of neurons in all layers of the cerebral cortex. We examined the alteration in neuronal morphology, including dendritic branching and the density of spines in neurons by Golgi-cox staining (Figure 5A). Dendritic density was markedly reduced in all experimental groups after MCAo, particularly the severe loss of dendritic connection and shorten neurite were observed in the pyramidal neurons throughout the cortex of Veh group (*p* < 0.01). In contrast, those changes were attenuated by treatment of MT and Ex, there was a significant increase of neurite length in MT, Ex and MT + Ex groups compared with Veh group from day 7 to 28 after MCAo (*p* < 0.01). In addition, MT + Ex group showed the dense of dendritic spines and progressive increase of crossing neurites at day 14 and 28 compared with both MT and Ex groups (*p* < 0.01). These results indicate that dual treatment of MT and Ex prevented the disruption of cortical pyramidal neurons and promoted the outgrowth of neurite (Figure 5B).

### 3.1 MT treatment improves cell viability and beating frequency

**Figure 6:**
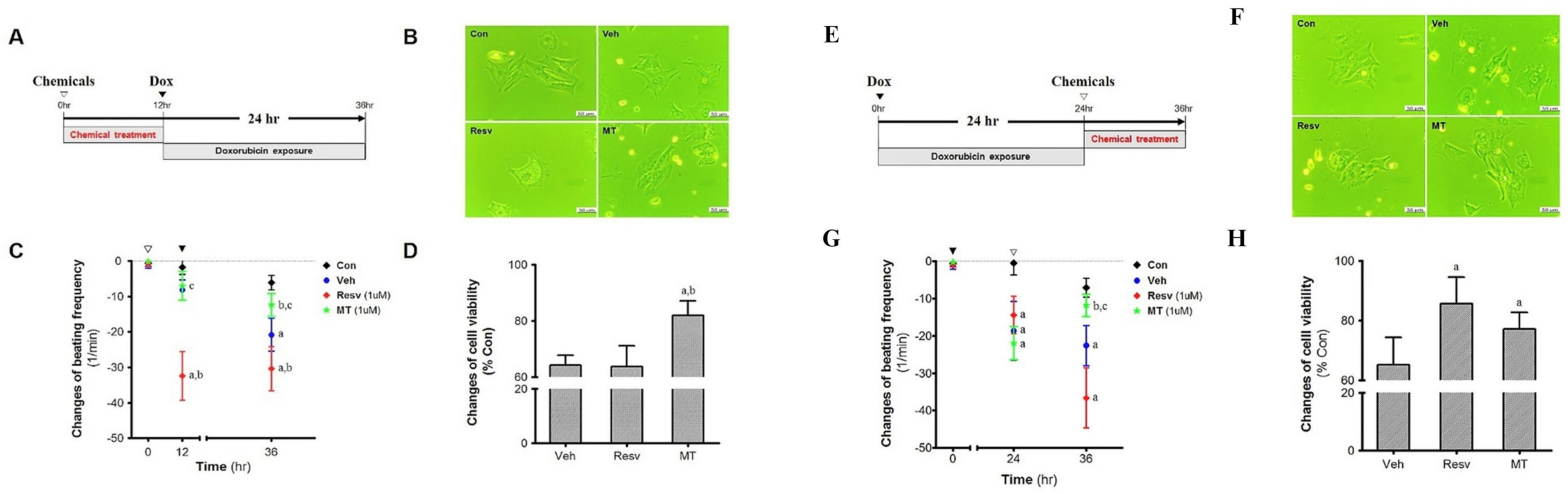
Pre-treatment effect of MT on the DIC. **(A)** Experimental scheme. **(B)** Morphology. **(C)** Cell viability, ^a^*p* < 0.05 vs. Veh; ^b^*p* < 0.05 vs. Resv. **(D)** Changes of beating frequency, ^a^*p* < 0.05 vs. Con; ^b^*p* < 0.05 vs. Veh; ^c^*p* < 0.05 vs. Resv. Post-treatment effect of MT on the DIC. **(E)**. Experimental scheme. **(F)**. Morphology. **(G)**. Cell viability, ^a^*p* < 0.05 vs. Veh; ^b^*p* < 0.05 vs. Resv. **(H)**. Changes of beating frequency, ^a^*p* < 0.05 vs. Con; ^b^*p* < 0.05 vs. Veh; ^c^*p* < 0.05 vs. Resv. Statistical significance was assessed by Kruskal–Wallis ANOVA and the Mann–Whitney *U*-test. Data shown are means ± S.E.M. Dox, doxorubicin; DIC, dox-induced cardiotoxicity; Ex, exercise; H.w., heart weight; MT, melatonin; T.l., tibia length; Veh, vehicle.

Both pre- and post-treatment of MT and Resv were performed with reference to Dox in primary neonatal cardiomyocytes. Many researchers have found that Resv may reduce Dox-induced cardiac toxicity by reducing reactive oxygen species (ROS)-induced damages. Therefore, Resv was used to compare the antioxidant potential of MT. In the pre-treatment group (Figure 6A), the microphotographs of the isolated cells in the control group showed a continuous morphology with spontaneous and synchronic beating (Figure 6B). As opposed to the control cells, partially contracting cardiomyocytes were observed in the Veh and Resv groups. Furthermore, several small vacuoles were found in cardiomyocytes cytoplasm in Resv treated group (Figure 6B). Similarly, after 12 h of incubation of cardiomyocytes with Resv, a very significant change in beating frequency was observed which is lower than both control (*p* < 0.05) and vehicle groups (*p* < 0.05) (Figure 6C). No statistically significant change was observed between MT, control, and Veh group prior to Dox treatment. As expected, after 24 h of the Dox-administration, the beating frequency in the Veh group was decreased as compared with the control group (*p* < 0.05). However, the change in beating frequency in Resv treated group remained unaltered after Dox treatment. In the MT group, the treatment of Dox showed an absence of statistical variance with the control group but a significant difference was observed with Resv (*p* < 0.05) and Veh (*p* < 0.05) groups (Figure 6C). Correspondingly, the cell viability was highest in the MT treated group which is higher than both Resv (*p* < 0.05) and Veh groups (Figure 6D).

In post-treatment groups (Figure 6E), control cells demonstrate similar morphology and beating pattern as the pre-treated control group (Figure 6F). Again, partially contracting and asynchronous cardiomyocytes were observed in the Veh and Resv group, while MT group was comparable to the control group (Figure 6D). A decline in beating frequency was observed in all the treated groups as compared with the control group after 24 h Dox treatment (*p* < 0.05) (Figure 6G). Furthermore, after 12 h of MT treatment, the beating frequency was enhanced and overlapped with the control beating frequency and significantly higher than the Resv and control group (*p* < 0.05). However, a further decline in the change in beating frequency was observed in Veh and Resv treated groups. It is worth noting that the decline in the Resv group was more pronounced than the Veh group (statistically insignificant though) and both groups remained lower than control (*p* < 0.05). Interestingly, the cell viability in MT and Resv groups were comparable, and both are higher than the Veh group (*p* < 0.05), indicating cytoprotective activity after DIC (Figure 6H).

### 3.2 Melatonin and exercise play a synergetic role in reducing Dox-induced cardiotoxicity in rats

**Figure 7:**
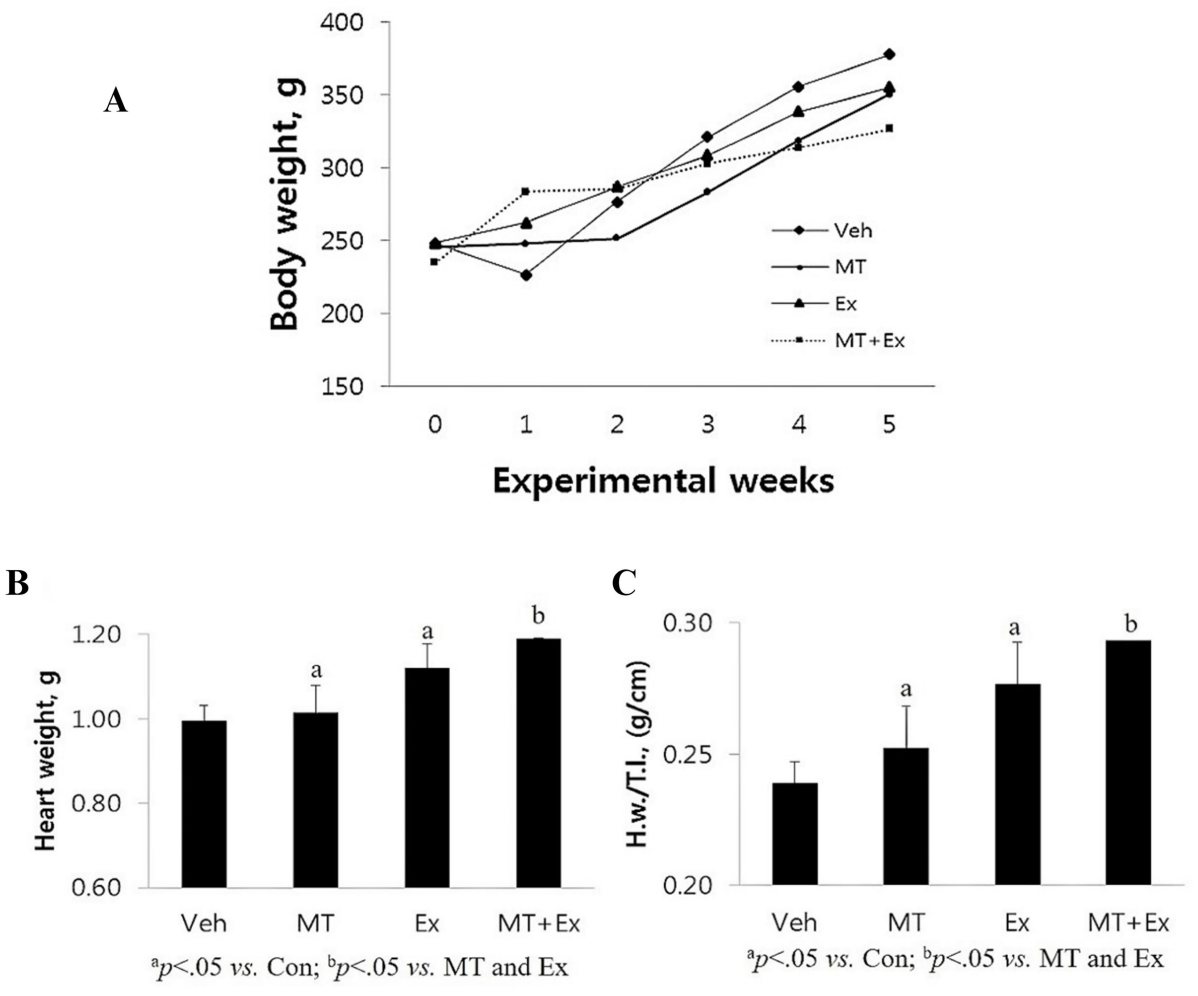
**(A)** Changes in body weight following the DIC and various intervention such as Veh, MT, Ex, and MT + Ex. Statistical significance was assessed by Kruskal–Wallis ANOVA and the Mann–Whitney *U*-test. No significant difference was found between the groups. **(B,C)** Changes in heart weight H.w. and H.w./T.l. following the DIC and various intervention such as Veh, MT, Ex, and MT + Ex. Statistical significance was assessed by Kruskal–Wallis ANOVA and the Mann–Whitney *U*-test. No significant difference was found between the groups. Data shown are means ± S.E.M. Dox, doxorubicin; DIC, dox-induced cardiotoxicity; Ex, exercise; H.w., heart weight; MT, melatonin; T.l., tibia length; Veh, vehicle.

After the first week of the experiment, the Veh group lost, MT + Ex group gained, and other groups remained approximately the same in body weight (Figure 7A). In the second week, all the groups gained weight except MT and MT + Ex groups. Subsequently, all groups of rats showed a similar gradual increase in body weight till the end of the experiments. Even though statistically insignificant, the vehicle group has the maximum weight on average at the end of the experimentation. Calabrese et al. (24) and Blanco et al. (25) exhibited that Dox supplementation in mice prevented weight gain. In our study, the vehicle and other groups did not show significant differences in body weight because the vehicle group rats each had 60–110 mL of ascites, which has already been reported to be associated with Dox-administration. Also, liver congestion and increased left ventricle size, which are heart-failure-associated symptoms, were observed in the Veh group. MT + Ex group has a minimum (statistically insignificant), while MT and Ex groups had similar body weight at the end of experiments (Figure 7B).

Variations in the animal body weight with ageing reduces the reliability of the body weight reference for normalizing heart weight (H.w.). Therefore, we have measured the H.w. and also normalized the H.w. by tibial length (H.w./T.l.) because the size of the tibia remains constant after maturity. Post experimentation analysis showed that Veh group had minimum H.w. as well as H.w./T.l. ratio amongst all the groups (*p* < 0.05) (Figure 7C), these results are similar to the results obtained in mice demonstrating that DIC caused a reduction in heart weight and left ventricle disorders. Both MT and Ex groups had similar H.w. and H.w./T.l., which was higher than Veh (*p* < 0.05), but lower than the MT + Ex group (*p* < 0.05).

### 3.3 Histopathological changes in the cardiac tissues

**Figure 8:**
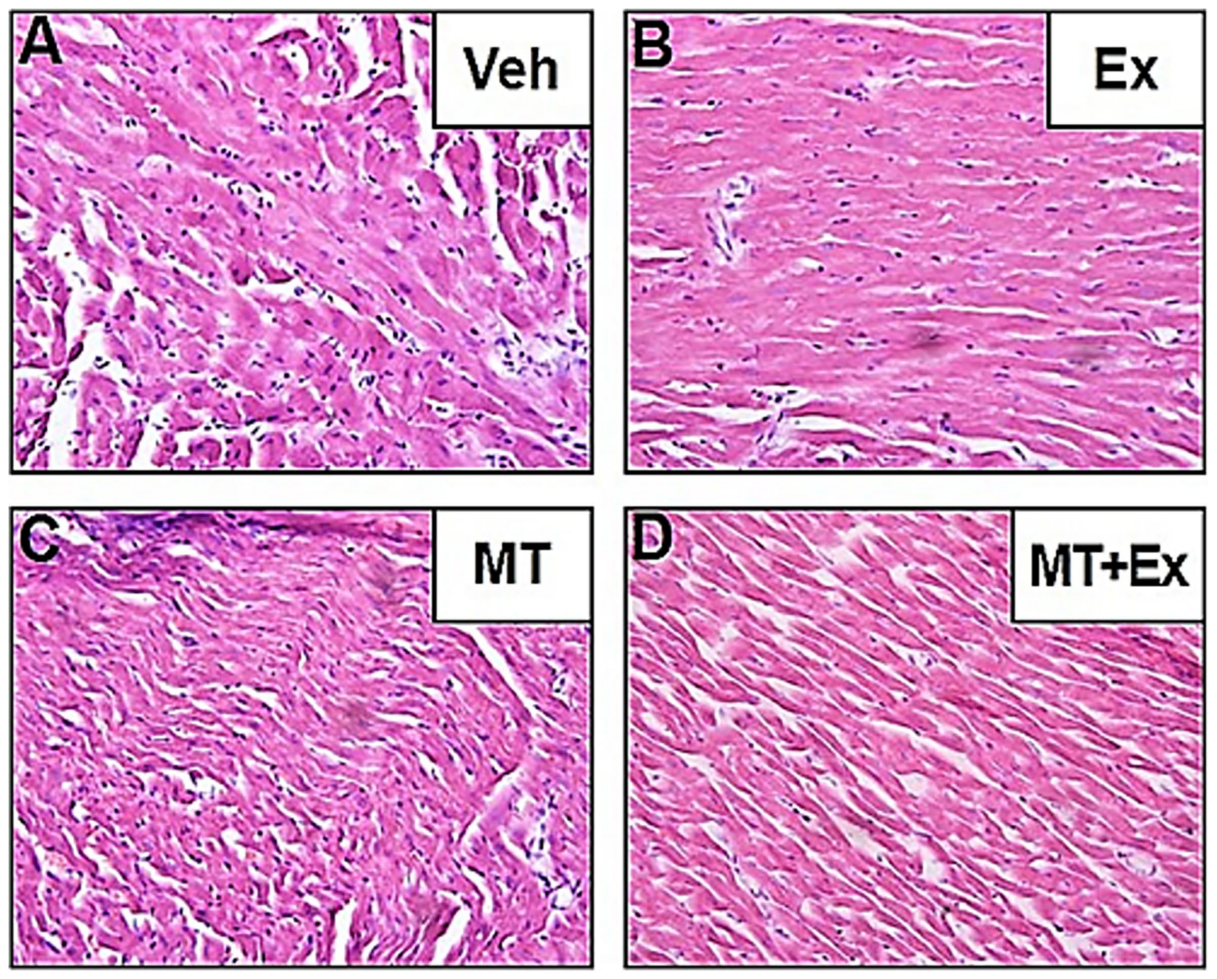
Histopathological changes induced by Dox and protective effect of MT, Ex, and MT + Ex in rat heart (H&E staining, magnification = 40×). **(A)** Cardiac section from Veh treated rats showing degenerative changes. **(B)** Cardiac sections from Dox-treated rats followed by Ex training. **(C)** Represents cardiac section from Dox-treated rats followed by MT administration. **(D)** Represents cardiac section from Dox-treated rats followed by MT + Ex treatment. Dox, doxorubicin; Ex, exercise; MT, melatonin; Veh, vehicle.

The HE staining displayed noteworthy histopathological changes in the rats of the Veh group as compared to those in rats of the MT, Ex, or MT + Ex groups. In the Veh group, some portion of the section appeared enlarged and exhibited degeneration, necrosis, myofibril twist, and myocardial fiber rupture (Figure 8A). Twisting and necrotic lesions were reduced in the MT and Ex groups as compared to the Veh group (Figures 8B,C). Most necrosis, myofibril twist, and degenerative lesions were absent in the MT + Ex group; individuals still showed a small amount of inflammatory cell infiltration (Figure 8D). The HE staining presented the protective effect of MT, Ex, and MT + Ex, later being the most prominent.

## 4 Discussion

Melatonin, a powerful an antioxidant, is neuroprotective and cardioprotective (26). It has been reported that MT, a neuroprotectant can influence neurogenesis, and neurodegeneration processes including ischemic brain injury (26). In this study, we found that combined therapy with MT and Ex could decrease the secondary damage associated with neural and cardiac injury and prevent the side effects related to Ex-induced fatigue and impairment. Therefore, we investigated the effects of exogenous MT plus treadmill Ex on functional recovery and regeneration of neural circuits after focal cerebral ischemia as well as after DIC.

This study considers both receptor-dependent and receptor-independent mode of action of MT that involve complex molecular crosstalk with other players, especially in ischemic injury (27) Although MT receptors may be downregulated in the morning, there is evidence to suggest that exogenous MT can still exert beneficial effects, particularly in pathological conditions associated with oxidative stress and inflammation (27, 28). The administration of MT was based on its potential to modulate inflammatory pathways and oxidative stress (NOS blot in manuscript) responses, which are known to be involved in stroke and cardiotoxicity.

We induced moderate-severe ischemic brain injury by 60-min middle cerebral artery occlusion. MCAo-induced animals exhibited hemiparesis symptoms including circling gait toward paretic side, flexion pattern of forelimb, and spasticity sign on balancing on the beam. Whereas attenuation of these neurological changes after MT and Ex treatment was observed in parallel to a delayed progression of the injury, showing significantly improved functional outcome mNSS score, as assessed the 4-week post-injury follow-up period. This is consistent with the TTC measurement of cerebral infarct volume showing reduction of the infarct expansion by MT and Ex.

We also demonstrated that ischemic brain injury induced neuronal damage and glial response by confirming the decrease of NeuN expression and overexpression of GFAP in the ischemic infarct region after MCAo. The activation of glia cells by ischemic injury has been evaluated by measuring the expression of GFAP, which is a biomarker synthesized by astrocytes in response to physical or metabolic injuries (29). Astrocytic responses are known as one of the earliest and most conspicuous changes in the CNS following ischemic injury (29). Furthermore, glial scar formation due to reactive astrogliosis declines the regenerative capacity after adult brain injury (30). Even though glial scarring increases the infarct size in the brain, it also has neuroprotective effect. It has been reported that astrocytes regulate and optimize the environment within which neurons act, also function to maintain the tight control of local ion and pH homeostasis, deliver the glucose, and provide the metabolic substrates (29, 30). Thus, there have been debates regarding the role of astrocytes in cerebral ischemia. Based on our result showing reduced GFAP expression through the dual treatment of MT and Ex, we suppose that glial scar formation is related to secondary neuronal damage.

Under physiological conditions, NO plays a key role as a neurotransmitter in CNS, however elevated levels of NO can promote tissue injury, including the development of secondary injury in ischemic brain damage (31). It is known that ischemia-induced NO is classified as a detrimental or beneficial influence on stroke. iNOS-provoked NO leads to caspase-mediated apoptosis, while eNOS-generated NO enhances to turn on the recovery machinery by microvascular vasodilation and angiogenesis (32). Emerging evidence implicates that MT has a neuroprotective effect, showing the elimination of excessive free radicals such as NO, superoxide, and hydroxide. Previously, we also have shown that both exogenous MT and treadmill Ex treatments eliminated iNOS levels in animal models of MCAo (33). Therefore, we determined that MT and Ex have a neuroprotective effect followed by the capability of free radical scavenger against free radical-derived secondary damage. However, there is little known about the alteration of nNOS associated with ischemic brain injury compared to the previous two NOS isoforms. We found that nNOS expression was significantly reduced after MCAo, particularly at the acute phase (3 days post-injury). Their expression then showed spontaneous recovery from day 7 after ischemic injury, which also matched with the expression pattern of NeuN. Recent studies suggest that nNOS, the major nitric oxide synthase isoform in the mammalian brain, is connected to the pathophysiology of several neurological conditions including cognitive dysfunction, Alzheimer’s, and epilepsy (34). Especially, nNOS is linked to hippocampal dentate gyrus (DG) neurogenesis in epileptic brain (34). Neurogenesis in the adult brain only occurs in the olfactory bulb (OB) and the hippocampus, and this is one of the obvious form of brain structural plasticity by which new functional neurons are generated from adult NSCs/NPCs (35). While NO produced by nNOS can exacerbate oxidative stress and neuronal injury in pathological conditions (36), our study demonstrates that moderate upregulation of nNOS following a stroke induced downregulation, as observed with the MT + Ex treatment, can activate neuroprotective signaling pathways (20). Specifically, optimum levels of nNOS-derived NO enhance cerebral blood flow (21), synaptic plasticity (22), and neuronal survival (24, 37), particularly when oxidative damage is counteracted by melatonin’s antioxidant properties and exercise-induced adaptive mechanisms (25). Thus, we reconcile the dual role of nNOS by showing that its protective effects emerge when the redox environment is optimized, with MT and Ex working synergistically to balance NO production, favoring its neuroprotective roles while minimizing oxidative damage (23). Selective silence of nNOS inhibited the proliferation and survival of hippocampal DG newborn cells, indicating that nNOS promotes adult hippocampal neurogenesis. In this study, the reduction of nNOS level by ischemic injury was restored by MT and Ex, additionally, MT combined with Ex therapy significantly reversed the expression of nNOS at the acute phase. Therefore, we consider that increased nNOS by MT plus Ex at the acute phase might be involved in neuroprotection and adult neurogenesis.

BDNF is one of a family of neurotrophic factors that influence neuronal proliferation, survival, and differentiation (38), as a result of binding to its tyrosine kinase receptor (Trk). Both BDNF and Trk are widely distributed throughout the brain, with the highest expression in the hippocampus (39). BDNF is stored and released from glutamate neurons in a use-dependent fashion and has been implicated in long-term potentiation (LTP), learning, memory formation, and cognition. It has been identified that BDNF plays as a regulator of various forms of neuroplasticity in neurodegenerative disease processes including focal ischemic brain injury, tau pathology, which contributes to AD (27). Furthermore, BDNF is also involved in metabolic growth factors to regulate adult neurogenesis, which occurs principally in the subventricular zone (SVZ) and in the hippocampal DG. We observed the two types of BDNF isoforms, pro-BDNF and mBDNF, which are identified with different signaling pathways. Pro-BDNF stimulates neuronal apoptosis through the p75 receptor, whereas mBDNF increases neural regeneration and rehabilitation of cognition and memory through TrkB (28). Though Pro-BDNF showed no significant difference, we considered that neural regeneration emerged by an alteration of mBDNF in the present study. mBDNF was upregulated not only in MT, EX, and MT + Ex groups but also Veh group. Thus, focal ischemic brain damage might result in a change of BDNF expression as a neuroplasticity action for spontaneous recovery.

Previous study has reported that exogenous MT could induce the differentiation of immature neuronal cells into mature neuronal cells in focal cerebral ischemia. To investigate the effects of MT and Ex on specific molecular markers of neurogenesis after ischemic brain injury, we thus confirmed the changes of DCX expression in MCAo rats. We found that DCX expression was significantly downregulated in vehicles treated MCAo rats at day 3 post-injury. Although the restoration of DCX was not detected in either MT- or Ex-treated rat, intervention of MT combined with Ex protected the DCX expression at the acute phase after damage.

There were degenerating morphological changes in pyramidal neurons after ischemic brain damage, including structural abnormalities and loss of dendritic spine density. However, intervention of MT and Ex enhanced the neural regeneration and functional recovery in our stroke animal model. We observed significant increases in dendritic spine density and neurite outgrowth in MT- and Ex-treated rats, particularly intervention of MT plus Ex ameliorated the dendritic impairment and neurite lengthening persistently from day 3 to 28 post-ischemic insult. A study by Juan et al. (40) as supporting results also showed that MT improved the dendritic arborizations in both cultured neurons exposed to glutamate excitotoxicity and MCAo rats through the upregulation of GAP-43, PSD-95, and MMP-9 expressions.

Although the previous study explored the role of MT and Ex individually with some success in the prevention of DIC (8). This study, in a first, showed the synergistic action of MT + Ex and attempted to directly compare the combined efficacy with the individual effect of Ex or MT intervention on DIC. This study showed that MT combined with Ex offers a synergistic protective effect against DIC and offers better protection than the MT or Ex, individually. Also, previous studies with MT and Ex intervention on DIC evaluated the biochemical and molecular parameters and claimed that protection against DIC was due to antioxidant activity including suppression of lipid peroxidation and NADPH oxidase 2 (NOX2) by MT and Ex, respectively (41). However, research on the functional aspects of cardiomyocytes for instance, cardiomyocyte beating was not performed that will provide direct clinical relevance.

Our *in vitro* results showed a protective effect of MT in both pre- and post-treatment conditions, but the pre-exposure of Resv did not improve cell viability. Besides, in both the pre- and post-treatment Resv treatment changes the morphology of myocardial cells and significantly reduces myocardial rhythm, suggesting that Resv is not a completely nontoxic candidate. On the other hand, in the case of MT, in addition to improving the cell viability, the heart muscle cell beat was similar to the control level until the end of the experiment. Unlike the pre-treated group, the post-treatment Resv significantly reduces Dox-induced cell death. This differential administration-dependent behavior of Resv might be due to the rapid metabolism of Resv and poor bioavailability leading to the absence of bioavailable Resv at the time of Dox administration in the pre-treatment group (42). Hence, higher concentrations or multiple dosages within a short duration are required to confirm the pre-treatment effect of Resv on DIC. Nonetheless, the number of myocardial beats further decreased following the Resv supplementation after the Dox-treatment. This decrease of myocardial beats with an improvement of cell viability by Resv treatment is extremely noteworthy and indicates the importance of studying functional aspects of myocytes like beating frequency. The Resv is known to have benefits as well as adverse effects as it may be cytoprotective or cytostatic depending upon the dose and exposure time (different role in day and night) (43, 44). However, simultaneous positive (improvement of cell viability) and negative (reduction in beating frequency) role within the same experiment was unknown, which is alarming, and warrants immediate research. On the other hand, MT improved the number of myocardial beats up to the control level. The comparative analysis of the obtained results showed that MT has no adverse effect on cardiomyocytes and offers superior cardioprotective activity than Resv in both the pre- and post-treatment, with higher protection efficacy in the post-treatment group. The results also suggest that as DIC is multifactorial, therefore, a multifaceted and biocompatible molecule such as MT should be explored in combination with other interventions rather than one-dimensional antioxidant only cytoprotectants. As we have observed better protective activity of MT against DIC in the post-treatment group, we have studied the post-treatment effect of MT + Ex and compared it with their individual protective action in rats.

In contrast to various studies that utilized supraclinical dosages (15–24 mg/kg) of Dox to explore the effect of therapies and pathways of DIC (44), which might give misleading results (especially in control group), we administered a clinically relevant Dox dose to mimic chemotherapy-associated cardiotoxicity. We observed that Dox not only induces cardiac toxicity-related heart failure symptoms but also heart failure-associated pathological symptoms such as liver congestion and ascites. The major goals of any therapy for chronic heart failure continue to be reducing left ventricle wall stress, increasing cardiac output, and reducing afterload. It is known from the past literature that Dox administration depress the body weight, reduced the H.w. and H.w./T.l. (45). In our study, the body weight difference would have been significant if the weight of Dox-induced ascites was subtracted from the total weight of the animals in the Veh group. Besides, H.w. and H.w./T.l. comparisons showed that Ex training and its combination with MT were effective. Additionally, we observed a loss of myofibers and striations, granular, fragmented cytoplasm, and cytoplasmic vacuolization in the Veh group. Treatment with MT and/or Ex training significantly reduced the loss of myofibers, loss of striations, granular, fragmented cytoplasm, and cytoplasmic vacuolization, and heart failure-associated pathologic symptoms (46–49). Although all of these treatments were effective, MT + Ex training had a greater protective effect on DIC, suggesting a synergistic effect. Furthermore, *in vitro* and animal experimental investigations have exhibited that MT could inhibit the growth of few human tumor models, MT may be very effective in alleviating cardiotoxicity and promoting antineoplastic activity of Dox.

In this study, we found that combining MT and Ex enhances neurological function recovery and reducing DIC. In the MCAo stroke model, the MT + Ex group showed significantly greater reductions in brain infarct volume and neurological deficits compared to either treatment alone, alongside enhanced neuronal growth and structural recovery. Similarly, in the DIC model, MT and Ex individually provided cardioprotection, but their combination led to superior outcomes in preventing heart weight loss and histopathological damage. These findings suggest that the interactions between MT and Ex are not merely additive but result in a more robust, multifaceted protective effect, highlighting their potential as a synergistic therapeutic strategy for addressing stroke and chemotherapy-induced cardiotoxicity While concurrently investigating the impact of stroke and DIC with a unified intervention is crucial for advancing research in this domain, a limitation of our study is the absence of a combined animal model of MCAo and DIC to uncover the pathway behind the synergistic protective effect of MT + Ex. We recognize the importance of including both male and female animals in preclinical research, our decision to focus on male rats was based on several factors, including the well-established endocrine associated sex differences. Future studies should aim to include both male and female animals to provide a more comprehensive understanding of the potential therapeutic effects of MT and Ex. Another limitation is the small sample size may limit the generalizability of the results and warrants further investigation with larger cohorts to confirm the findings. Also, future studies may incorporate individualized exercise testing, such as lactate threshold or VO₂ max assessments, to refine exercise dosing and further enhance translational relevance.

## Data Availability

The original contributions presented in the study are included in the article/Supplementary material, further inquiries can be directed to the corresponding author.
