# Supplementary material for: Synergistic benefits of melatonin and therapeutic exercise as a unified strategy for managing stroke and doxorubicin-induced cardiotoxicity
Source: Front Neurol. 2025 Nov 5;16:1567263. doi: 10.3389/fneur.2025.1567263 (PMC12627009; doi:10.3389/fneur.2025.1567263)

Neun & GFAP

Left to right- Con, Veh, MT, Ex, MT +Ex and replicates

NeuN (MAB377, Millipore)

- 46/48 kDa
- Dilute to 1:1000
- 2nd antibody (Mouse) was diluted to 1:5000

GFAP (#3670, Cell Signaling)

- 50 kDa
- Dilute to 1:2000 and 1:4000
- 2nd antibody (Mouse) was diluted to 1:5000
- Used 2.5% BSA for Blocking, 1st and 2nd ab

Day 3

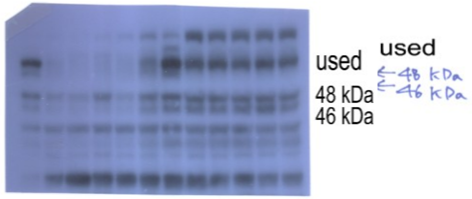

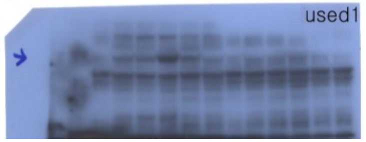

Day 7

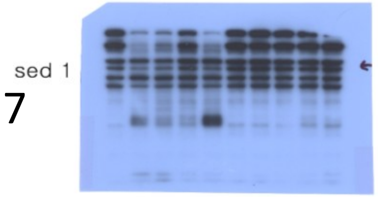

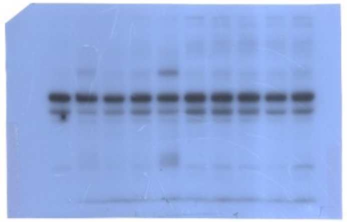

Day 14

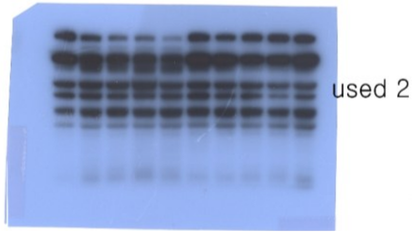

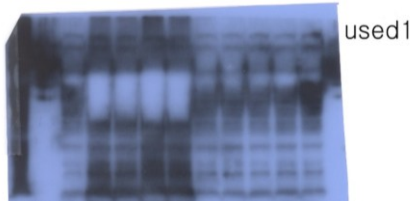

Day 28

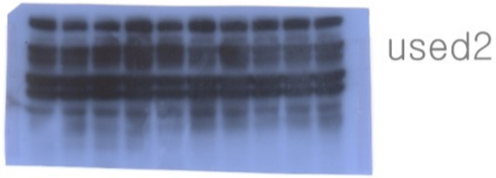

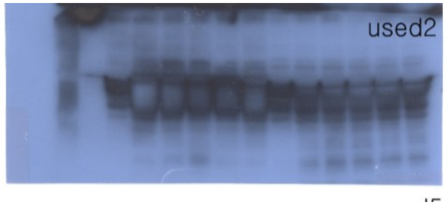

nNOS & BDNF

Left to right- Con, Veh, MT, Ex, MT +Ex and replicates

nNOS (#2982, Cell Signaling)

- 130 kDa
- Dilute to 1:1000
- 2nd antibody (Rabbit) was diluted to 1:5000

BDNF (SC 33904)

- Precursor 30 kDa, Mature form 14 kDa
- Dilute to 1:1000 New and Old (made on Aug)
- 2nd antibody (Goat) was diluted to 1:5000
- Used 2.5% BSA for Blocking, 1st and 2nd ab

Day 3

used

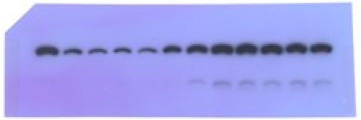

used 1

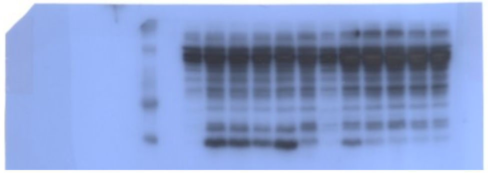

Day 7

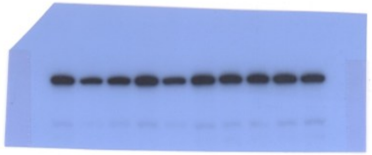

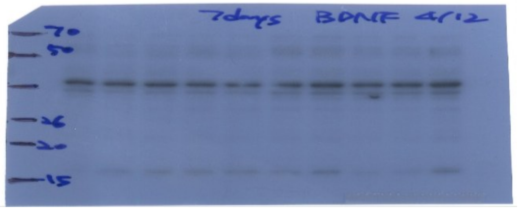

Day 14

used 4

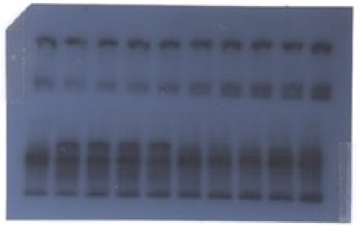

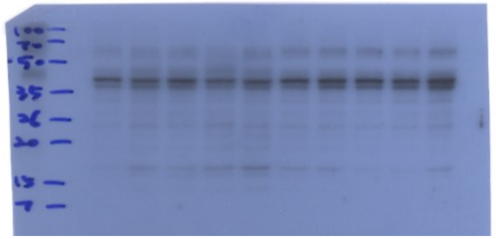

Day 28

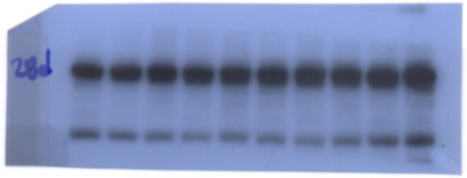

used 1

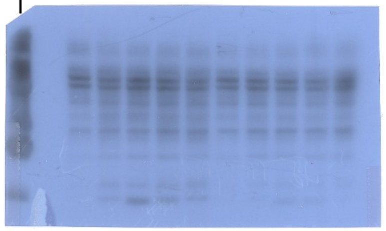

Doublecortin (DCx) (SC8066)

- 40 kDa
- 2.5% BSA was used for blocking, 1st and 2nd ab'
- Dilute to 1:1000
- 2nd antibody (Goat) was diluted to 1:5,000

Day 3

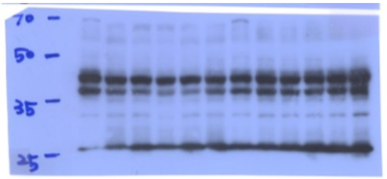

Day 7

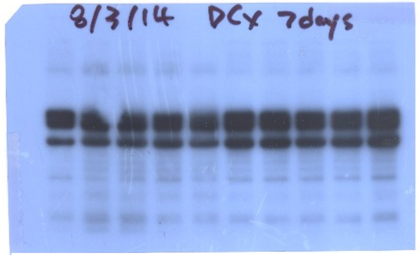

Day 14

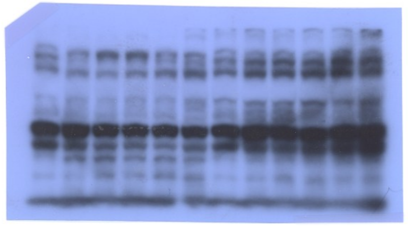

Day 28

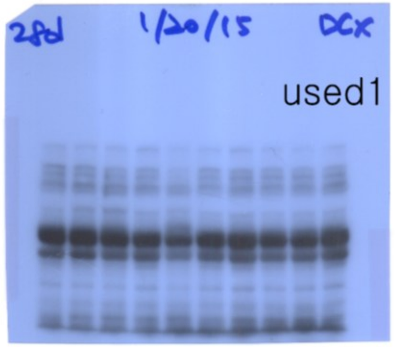

Left to right- Con, Veh, MT, Ex, MT +Ex and replicates

Day 3 GAPDH

Left to right- Con, Veh, MT, Ex, MT +Ex and replicates

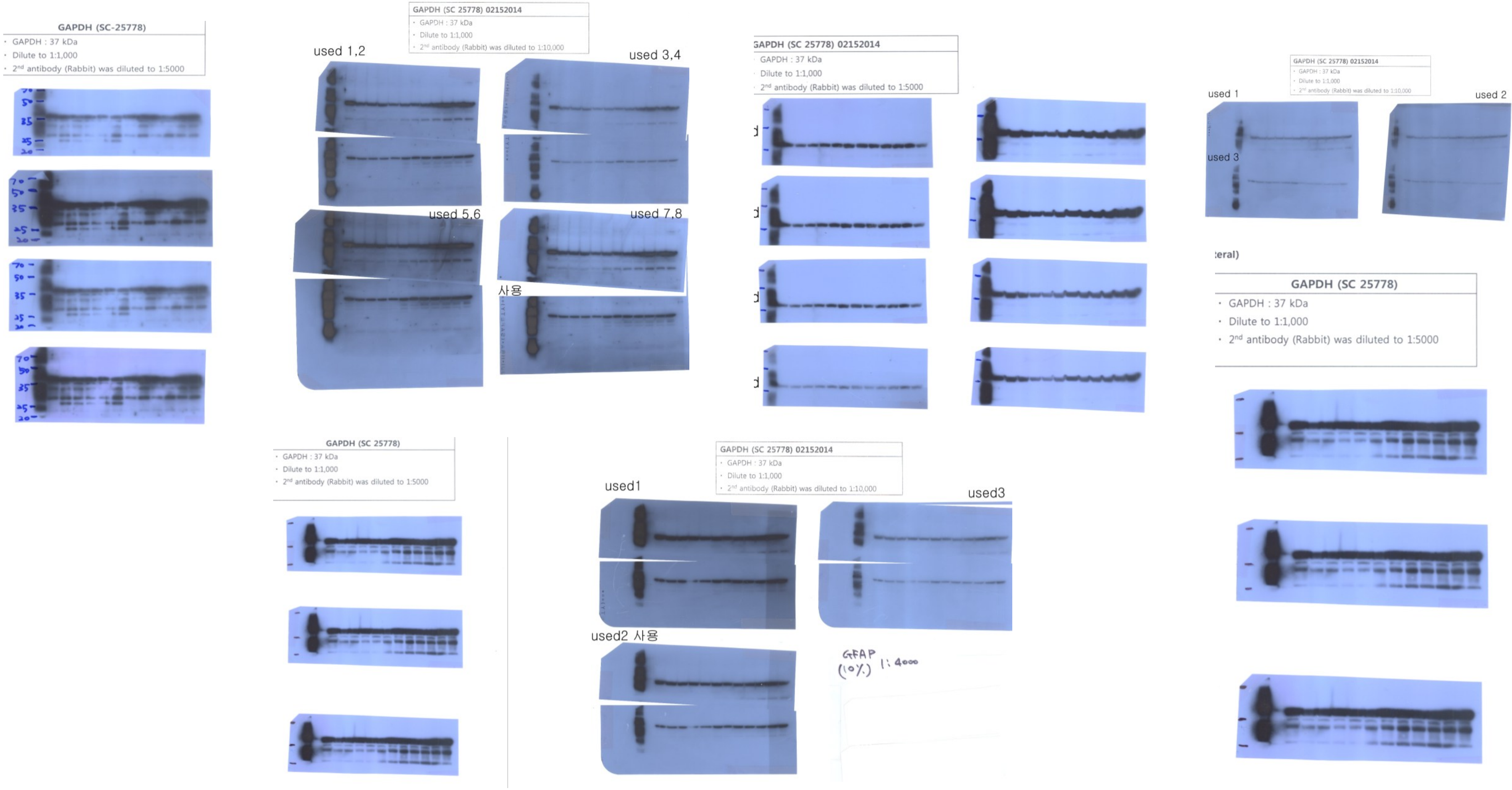

Day 7 Gapdh

Left to right- Con, Veh, MT, Ex, MT +Ex and replicates

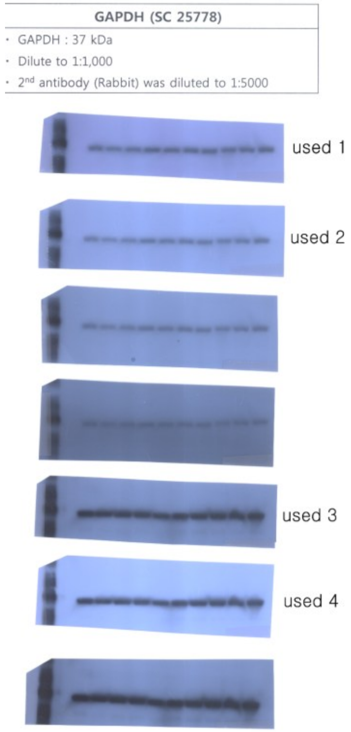

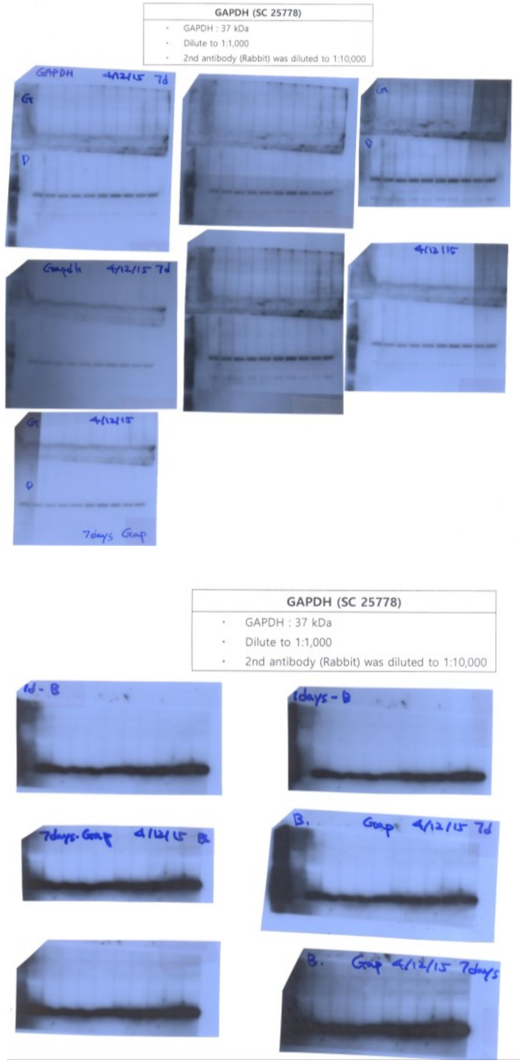

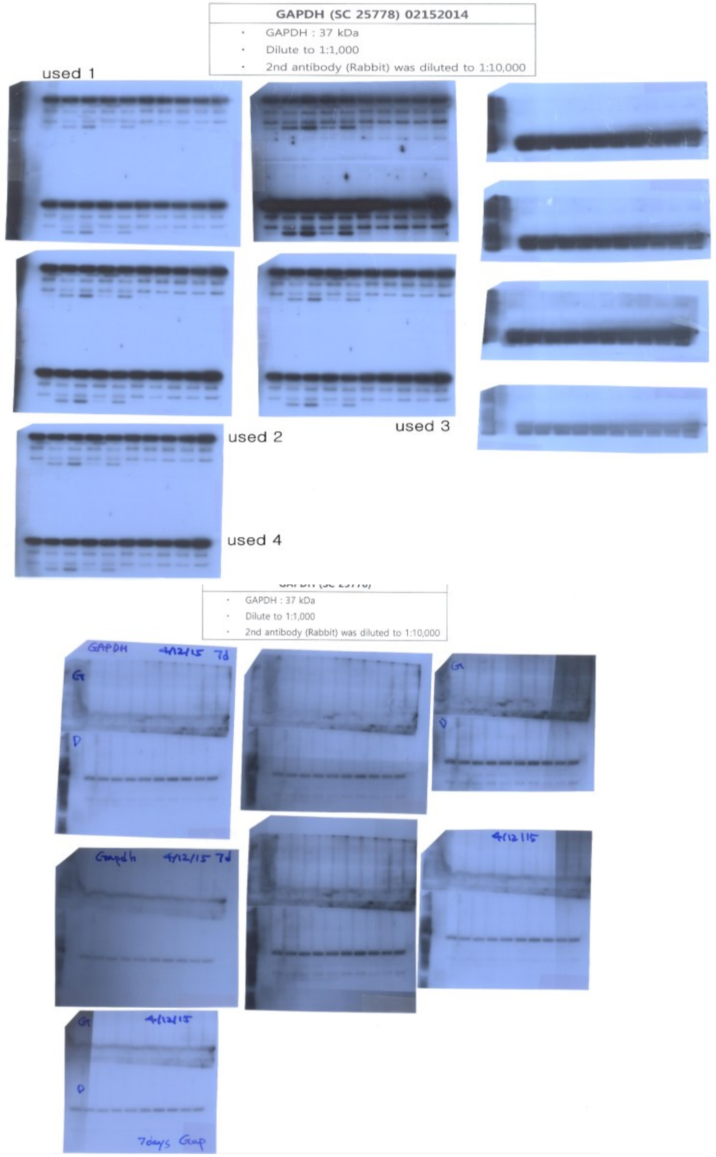

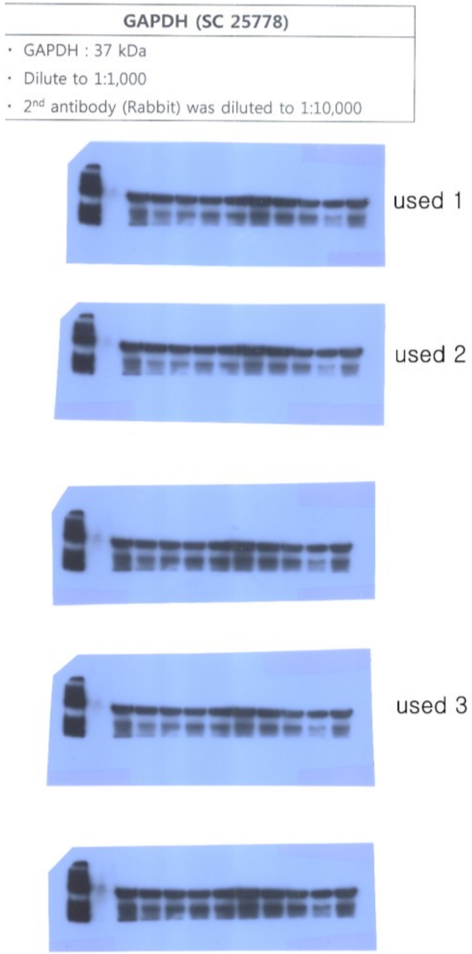

Day 14 Gapdh
Left to right- Con, Veh, MT, Ex, MT +Ex and replicates

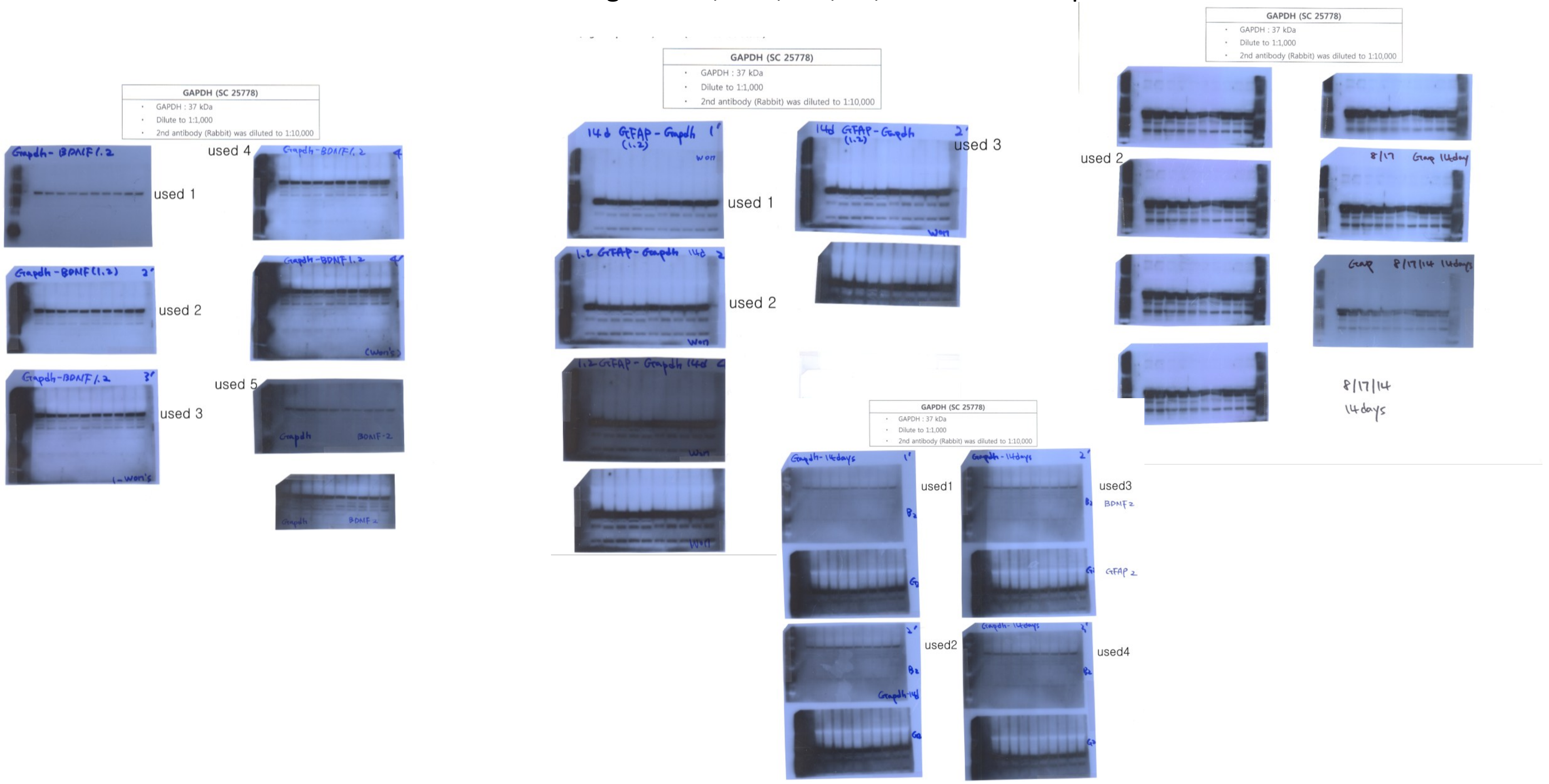

Gapdh day 28

Left to right- Con, Veh, MT, Ex, MT +Ex and replicates

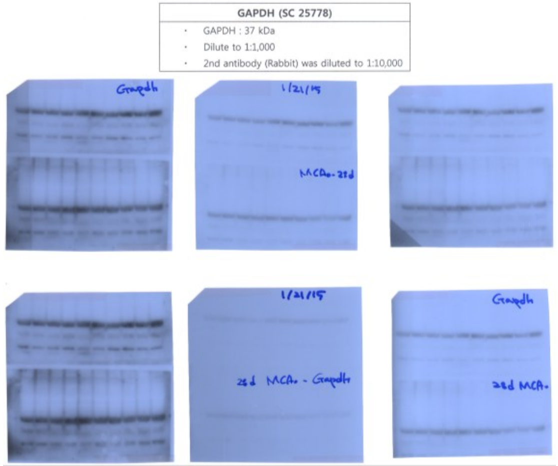

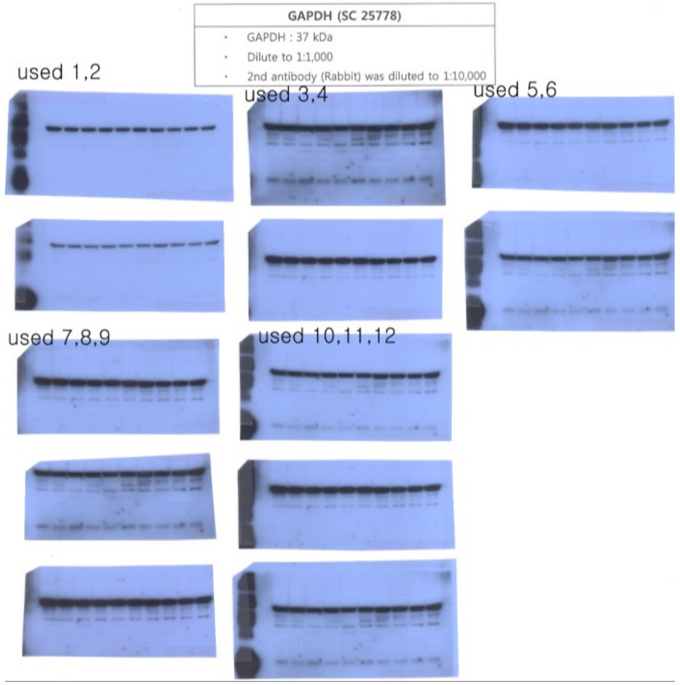

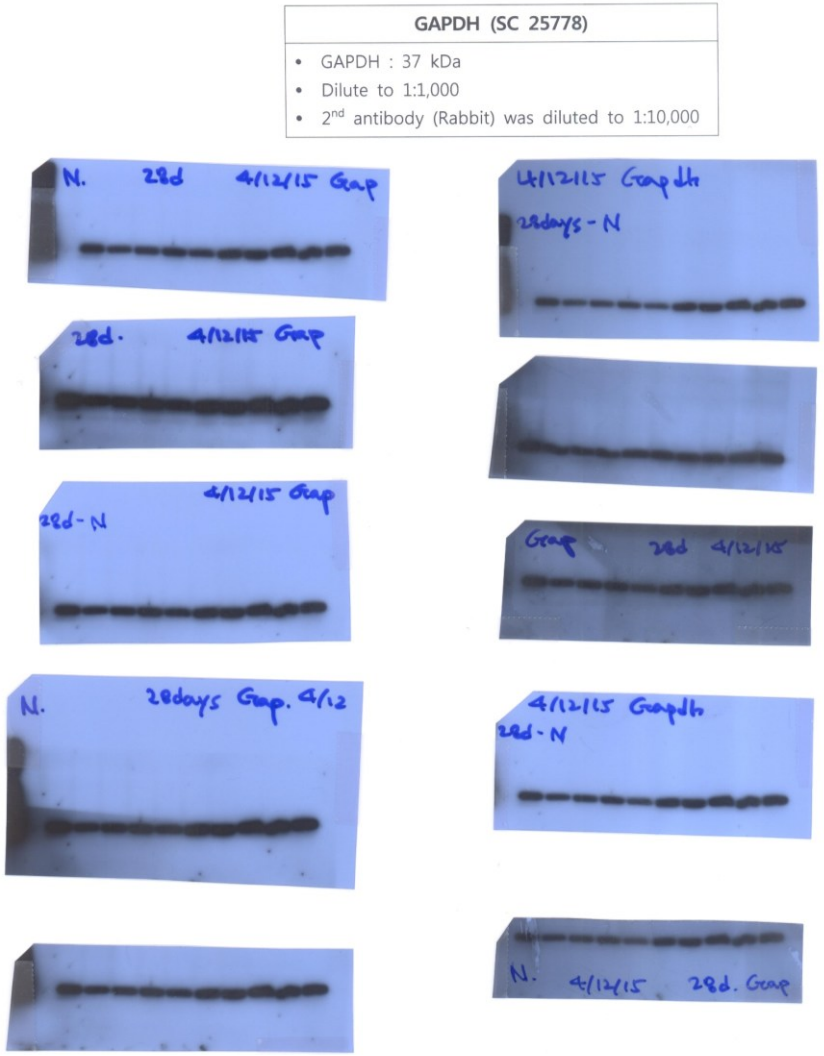

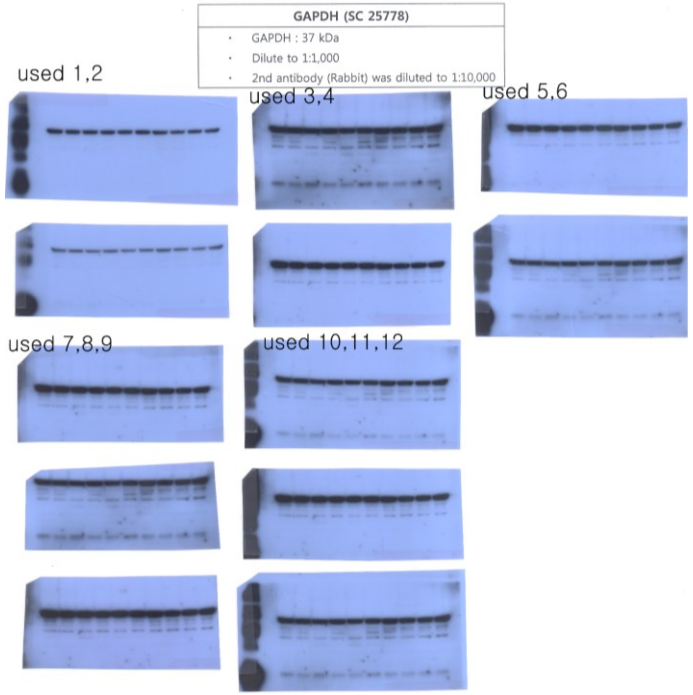

Supplement: Supplementary file 1 [file Data_Sheet_1.PDF]
